# Dynamic visual acuity in bilateral vestibulopathy and healthy age–sex-matched participants

**DOI:** 10.1007/s00415-025-13269-9

**Published:** 2025-09-01

**Authors:** Meichan Zhu, Lisa van Stiphout, Benjamin Volpe, Miranda Janssen, Mustafa Karabulut, Angélica Pérez Fornos, Nils Guinand, Kenneth Meijer, Raymond van de Berg, Christopher McCrum

**Affiliations:** 1 Department of Otorhinolaryngology and Head and Neck Surgery, Division of Balance Disorders, School for Mental Health and Neuroscience, Maastricht University Medical Center, Maastricht, The Netherlands; 2 Department of Nutrition and Movement Sciences, NUTRIM Institute of Nutrition and Translational Research in Metabolism, Maastricht University, Maastricht, The Netherlands; 3 Department of Otorhinolaryngology, Guangzhou Twelfth People’s Hospital (Guangzhou Otolarynology-Head and Neck Surgery Hospital), No. 1 Tianqiang Road, Tianhe District, Guangzhou, 510620 Guangdong China; 4 Department of Methodology and Statistics, Care and Public Health Research Institute (CAPHRI), Maastricht University, Maastricht, The Netherlands; 5 Service of Otorhinolaryngology and Head and Neck Surgery, Department of Clinical Neurosciences, Geneva University Hospitals, Geneva, Switzerland

**Keywords:** Bilateral vestibulopathy, Dynamic visual acuity, Oscillopsia, Walking

## Abstract

**Objectives:**

Dynamic visual acuity (DVA) can be assessed on a treadmill while walking at different speeds and is used to assess people with bilateral vestibulopathy (BVP). However, the effects and interactions of age, BVP, and walking speed on DVA loss and assessment dropout are unclear. Our objective was to investigate the effects of BVP, age, and walking speed on DVA loss and assessment dropout in participants with BVP and healthy age–sex-matched participants.

**Methods:**

41 participants with BVP and 41 age–sex-matched healthy participants completed a treadmill-based DVA assessment, including a static condition at 0 km/h and walking conditions at 2, 4, and 6 km/h. DVA loss was measured as the visual acuity difference between static and walking conditions. The drop-out rate, handrail use, and DVA loss were examined in relation to BVP, age, and walking speed.

**Results:**

Age significantly increased the odds of dropping out (odds ratio = 1.160, *p* < 0.001), while BVP did not increase the odds of dropping out (odds ratio = 0.792, *p* = 0.733). A significant Group*Speed (*p* = 0.004) interaction effect was found for DVA loss, with DVA loss being significantly worse in people with BVP across all walking speeds, getting progressively worse as speed increased, which was not seen in the healthy participants. Age did not have a significant effect on DVA loss (*p* = 0.06).

**Conclusion:**

BVP does not appear to restrict the ability to walk at the higher speeds of a DVA assessment and cause an increase in dropout rate, whereas age does. BVP significantly impacts DVA, with increasing impact at increasing walking speeds.

**Supplementary Information:**

The online version contains supplementary material available at 10.1007/s00415-025-13269-9.

## Introduction

Bilateral vestibulopathy (BVP) [1, 2] is a chronic vestibular syndrome characterized by unsteadiness during standing and walking (particularly in darkness, on uneven ground and during head motion) [3–6], as well as movement-induced blurred vision or oscillopsia [3–5, 7, 8], falls [9], compromised cognition [10], impaired spatial memory [11, 12], and reduced quality of life [10, 13]. Dynamic Visual Acuity (DVA) testing measures the functional result of gaze stabilization [14]. DVA assessment assesses the perception and identification of visual targets during passive (i.e., assessor-applied) [14, 15] or walking-induced head movements [16–20]. People with unilateral vestibulopathy or BVP have more DVA loss (DVAL) than healthy people during walking [17, 19]. Therefore, DVA assessment could contribute to screening for vestibular disorders [7, 17, 21].

Guinand et al. [17] demonstrated a rise in DVA test sensitivity for BVP with increasing walking speed: from 76% at 2 km/h to 97% when combining 2, 4, and 6 km/h. However, the downside is that some people with BVP may be unable to complete all walking speeds, leading to a speed-related dropout [17]. Starkov et al. [19] confirmed that BVP was significantly related to an increased dropout rate compared to healthy participants. This has important implications for treadmill-based DVA as an outcome measure for therapeutic interventions, such as a vestibular implant [22].

To fully understand DVA in people with BVP, the possible extent of the deficit in DVA due to aging itself, versus due to BVP, should be determined. As vestibular function naturally deteriorates with age [23, 24], older people may experience (sub-clinical) reductions in DVA and balance. Additionally, there is evidence of an age-related decline in DVA in people who demonstrate no vestibular deficit in caloric, rotary chair, and vestibular autorotation testing [25]. Guinand et al. [17] also reported an age effect on DVAL in healthy adults walking at 4 km/h and 6 km/h, despite normal head impulse test results.

Guinand et al. [17] and Starkov et al. [19] reported higher DVAL at all speeds in people with BVP compared to healthy participants. However, those healthy participants were not individually age-matched to the participants with BVP (in Guinand et al. [17], they were matched at group level and in Starkov et al. [19] they were, on average, 13 years younger) and neither set of participants was sex-matched. Therefore, it is unclear how much of the differences observed might be attributable to these group differences.

The objective of this study was to investigate the effects of BVP, age, and walking speed on DVAL and DVA assessment dropout, by comparing these outcomes between participants with BVP and healthy age–sex-matched participants. It was hypothesized that both BVP and age would be significantly associated with the dropout rate and that participants with BVP would experience more DVAL than healthy age–sex-matched participants.

## Methods

### Participants and study design

Between June 2021 and June 2022, 52 participants with BVP were enrolled in a large cross-sectional study of BVP at Maastricht University Medical Centre, a tertiary referral center. Given the relative rarity of the disease, no a priori sample size calculation was performed. Rather, we invited all patients previously diagnosed with BVP in Maastricht University Medical Centre to participate in the large cross-sectional study. Inclusion criteria aligned with the BVP diagnostic criteria of the Bárány Society's Classification Committee [1, 2]. Individuals with polyneuropathy, those unable to discontinue vestibulo-suppressive medication, unable to sit in the testing chair for an hour, unwilling to undergo detailed physical or vestibular examinations, or younger than 18 were excluded. If a participant could not walk at 2 km/h on the treadmill or if their vision was ≤ −4.0 diopters (without corrective lenses), their DVA was not assessed. All participants provided written informed consent prior to participation. The study was carried out in accordance with the Declaration of Helsinki, and it was given approval by the azM/UM Medical Ethical Committee (METC: NL72200.068.19). Once enrolled, participants’ diagnoses of BVP were confirmed using the Bárány criteria [1, 2] (see eMethods in Supplement 1). Previous reports of analyses of other outcomes of the larger study can be found elsewhere [26, 27]. Note that this was the same patient cohort as reported in the study of Starkov et al. [19].

Between February 2023 and August 2024, 52 healthy age–sex-matched participants were enrolled in a second data collection period to form a well-controlled reference dataset for the larger BVP study. The inclusion criteria were: self-reporting as healthy without known vestibular or neurological symptoms or disorders, psychiatric conditions, or chronic drug/medication use (recreational or medical), and within ± 2 years of age of an included participant with BVP of the same sex. The use of alcohol or other stimulants was prohibited for 24 h before the assessment. Participants were recruited via posters distributed around the hospital, university, and city center, via social media and our department website for study recruitment (https://www.testheldmaastricht.nl/), as well as by word of mouth. All participants provided written informed consent prior to participation. This data collection was carried out in accordance with the Declaration of Helsinki, and it was given approval by the FHML Research Ethics Committee of Maastricht University (FHML-REC/2023/005).

### Setup and DVA assessment

Visual acuity was assessed by displaying Sloan optotypes (C, D, H, K, N, O, R, S, V, and Z) [28] on a computer screen (LG 24bk55 24″), generated by a custom Matlab application (R2022a; The Mathworks, Natick, USA) controlled via another screen. The display screen was positioned at eye level, 2.8 m away from the participant. Participants were instructed to identify and name the letters as they appeared. Each measurement started with a font size of 1.0 logMAR (logarithm of Minimal Angle of Resolution; lower logMAR indicates smaller letters and therefore better visual acuity) and an adaptive, two-down one-up staircase procedure based on participants’ responses was used to determine the size of the next optotype [29, 30]. Each optotype was displayed for one second and the researcher recorded if the response was correct or incorrect, which triggered the next optotype. Specifically, after two correct responses, the logMAR was reduced by 0.3 until the participant gave an incorrect response, which triggered a reversal to increase the logMAR by 0.3. After two consecutive correct responses, the second reversal was triggered, after which the logMAR adjustment was reduced to 0.2. This continued until the 5th reversal after which the logMAR was adjusted by 0.1. After 15–19 reversals, the test was concluded, and the visual acuity was calculated by taking the mean of the last ten reversal points. The participants completed the DVA assessment on the treadmill (1210 model, SportsArt, Inc., Tainan, Taiwan), with assessments at static (0 km/h) and walking conditions (2 km/h, 4 km/h, and 6 km/h [17]). The assessment ended after all walking speeds were finished or if participants were unable to perform the task at the required speed. If participants were unable to complete a specific walking speed, they were classified as a “dropout” for that speed. The dropout rate, which was the number of participants with a dropout divided by the total number of participants in the group, was used for analysis. DVAL was calculated as the logMAR of the static condition minus the logMAR of the dynamic conditions [31] and thereby a negative value indicates reduced visual acuity. A safety cord was attached to the treadmill's emergency brake and fastened at the participants’ waists. In some circumstances (anxiety or unsteadiness), participants with BVP were allowed to grip the treadmill handrails, though they were initially discouraged from doing so if possible.

### Statistical analyses

Ordinal logistic regression analysis was used to examine the age and BVP effects on dropout rate within each group. Multilevel logistic regression of dropout (yes: dropout at 4 km/h or dropout at 6 km/h or no: completed DVA at every speed) on age and group failed (due to multicollinearity). Therefore, a new dependent variable reflecting the missing pattern was created using the following criteria: (1): no dropout at any speed; (2): dropout at 6 km/h; and (3): dropout at 4 and 6 km/h. There was no dropout at 2 km/h since inability to walk at 2 km/h was an exclusion criterion.

To assess DVAL difference between participants with BVP and healthy age–sex-matched participants, and effects of group, age, and speed when testing DVA on a treadmill, marginal linear regression analyses were performed. Firstly, the most appropriate covariance structure of the residuals was determined. Three different models were compared: (1) a marginal linear regression model with an unstructured covariance matrix of the residuals; (2) a marginal linear regression model with a compound symmetry covariance matrix of the residuals with heterogenous variances; and (3) a marginal linear regression model with a compound symmetry covariance matrix of the residuals with homogenous variances (this equals a linear mixed model with a random intercept per participant). Models were compared using the likelihood ratio test (LRT), Akaike Information Criterion (AIC) and Bayesian Information Criterion (BIC). The fixed part of these models included group (BVP, healthy) and speed (2 km/h, 4 km/h, and 6 km/h) as factors and age as a covariate. In addition, all two- and three-way interactions were included. Secondly, non-significant interactions were removed from the fixed part of the model. Pairwise comparisons were made within each group to compare DVAL at 2, 4, and 6 km/h. Pairwise comparisons were also made per speed to compare DVAL between participants with BVP and healthy age–sex-matched participants.

To assess whether gripping the treadmill handrails with one or both hands affected DVAL, a marginal model with an unstructured covariance matrix of the residuals was used. DVAL was the outcome at any given speed point in BVP. Main effects included handrail use, speed, and an interaction between group and speed (Group*Speed).

Independent t-tests were used to compare the two groups in terms of age, height, body weight, and BMI. Analyses were performed using SPSS (v.27, IBM SPSS Statistics for Windows, NY). *P* < 0.05 was considered statistically significant. P values from multiple testing were adjusted using Holm– Bonferroni corrections. Figures were generated using GraphPad Prism 9 (GraphPad Software, USA).

## Results

### Participants

Fifty-two participants with BVP and 52 healthy age–sex-matched volunteers participated. On retesting, two participants with BVP no longer met Bárány classification criteria and were excluded. Four participants with BVP had no recorded DVA data (three due to improper file storage, one due to network failure while recording), and five participants with BVP were missing data from the static DVA measurement due to data saving errors. As a result, 41 participants with BVP and their corresponding 41 healthy age–sex-matched participants were included in the current analysis (demographic data in Table 1). The BVP etiologies and descriptive data from the vHIT, caloric, and torsion swing tests are displayed in eResults and eTable S1 in Supplement 1.

**Table 1:** Participant demographic data

	BVP	Range	N	Healthy	N	Range	t(df)	*P*
Age, years	57.8 (12.0)	25–77	41	57.7 (12.2)	41	25–77	t(80) = 0.10	0.920
Male (%)	53.6%		22	53.6%	22			
Height, cm	172.5 (8.9)	152.0–196.0	41	172.1 (9.6)	41	154.4–202.5	t(80) = 0.20	0.839
Body weight, kg	84.2 (21.8)	40.1–147.0	41	72.6 (10.0)	41	57.0–104.0	t(80) = 3.11	0.003
BMI	28.0(6.4)	17.4–47.7	41	24.5 (2.6)	41	19.3–30.7	t(80) = 3.20	0.002

Values are mean (SD)

### Dropout rate

Ordinal logistic regression revealed that age significantly increased the odds of dropping out at speeds of 4 km/h and 6 km/h (odds ratio = 1.160, *p* < 0.001, 95% CI: 1.061–1.268). BVP did not increase the odds of dropping out (odds ratio = 0.792, *p* = 0.733, 95%CI: 0.207–3.029). A breakdown of the dropouts per age group can be found in Supplement 1 (eTable S2).

### Dynamic visual acuity loss

**Fig. 1:**
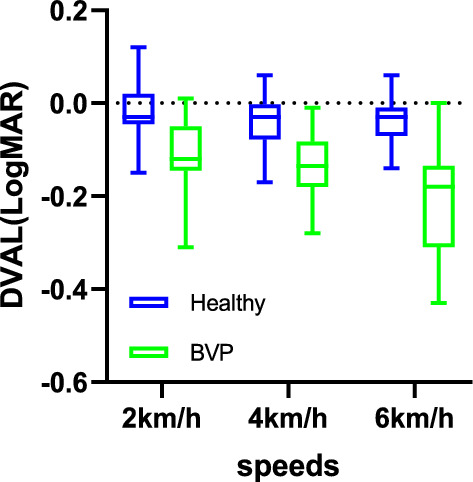
DVAL at three walking speeds in participants with BVP (green) and healthy age–sex-matched participants (blue). Box: lower quartile, median, upper quartile. Whiskers: minimum and maximum values

The model building, as described above, resulted in a marginal model with an unstructured covariance matrix of the residuals. DVAL was the outcome at any given speed point in both groups. Main effects included group, age, speed, and an interaction between group and speed (Group*Speed). This revealed a significant Group*Speed interaction effect (F_2,69.96_ = 6.052, *p* = 0.004) on DVAL (Fig. 1). Post hoc analyses revealed that participants with BVP experienced significantly more DVAL compared to healthy age–sex-matched participants at all walking speeds (*p* < 0.001; Table 2) and that speed significantly influenced DVAL in the BVP patient group (*p* < 0.001; Table 2). However, there were no significant post hoc comparisons for DVAL across the different walking speeds in the healthy age–sex-matched participants (*p* > 0.05; Table 2). Age did not have a significant effect on the DVAL (F_1,75.97_ = 3.643, *p* = 0.06).

**Table 2:** Post hoc comparisons for the Group*Speed interaction effect on DVAL

Group	Speed	Group	Speed	t	Df	P	Significant after Holm–Bonferroni Correction	95%CI
Healthy	2 km/h	Healthy	4 km/h	2.173	75.011	0.033	No	0.002–0.039
Healthy	2 km/h	Healthy	6 km/h	0.862	66.009	0.392	No	−0.019 to 0.047
Healthy	4 km/h	Healthy	6 km/h	0.447	65.985	0.656	No	−0.022 to 0.035
BVP	2 km/h	BVP	4 km/h	3.393	75.955	<.001	Yes	0.013–0.051
BVP	2 km/h	BVP	6 km/h	5.565	66.820	<.001	Yes	0.059–0.126
BVP	4 km/h	BVP	6 km/h	4.169	67.043	<.001	Yes	0.031–0.089
BVP	2 km/h	Healthy	2 km/h	6.408	71.814	<.001	Yes	0.121–0.230
BVP	4 km/h	Healthy	4 km/h	6.457	64.997	<.001	Yes	0.105–0.198
BVP	6 km/h	Healthy	6 km/h	7.547	69.498	<.001	Yes	0.120–0.206

### Handrail use

Among the participants with BVP, 22 (mean 49.91 years) out of 41 did not grip the handrail. 8 (mean 66.75 years) out of 41 used one hand to grip the handrail, while 13 (mean 68.31 years) out of 41 used both hands to grip the handrail. A marginal model with an unstructured covariance matrix of the residuals revealed significantly less DVAL across speeds in participants with BVP who did (one or two hands) versus did not hold the handrails (F_2,35.029_ = 5.574, *p* = 0.008). Pairwise comparisons (Table 3) indicated significant differences in DVAL between with and without handrail use at speeds of 4 km/h (*p* = 0.003) and 6 km/h (*p* = 0.017). Specifically, the DVAL was estimated to be on average 0.094 greater among participants without handrail use compared to with handrail use at 6 km/h and 0.118 greater at 4 km/h. No significant difference was observed at 2 km/h (*p* = 0.136; Table 3). Due to the significant impact of handrail use on DVAL, we decided to repeat our analyses with only the participants with BVP who did not use the handrails and their corresponding healthy age–sex-matched participants. The full results of these analyses can be found in eResults, eFigure S1, and eTables S3-S7 in Supplement 1.

**Table 3:** Post hoc comparisons of handrail effect at three different speeds

Group	Speed	t	Df	P	Significant after Holm–Bonferroni Correction	95%CI
BVP	2 km/h	1.518	43.696	0.136	No	−0.023 to 0.165
BVP	4 km/h	3.201	33.025	0.003	Yes	0.043–0.192
BVP	6 km/h	2.498	37.832	0.017	Yes	0.018–0.171

## Discussion

In this study, contrary to our hypothesis, age was the only factor significantly linked to increased dropout rates, suggesting that older individuals, regardless of vestibular function, find it more challenging to complete DVA assessments at faster walking speeds. We found a significant group by speed interaction effect on DVAL, with participants with BVP experiencing more DVAL as speed increased compared to healthy age–sex-matched participants, confirming our hypothesis. However, we did not find a significant interaction in the post hoc analysis of the participants who did not use the handrails, while group and speed were significant factors (eResults in Supplement 1), potentially due to a drop in statistical power, since eFigure S1 does indicate a steeper loss in DVA in the participants with BVP.

Our dropout rate findings differ compared to our previous study [19] conducted in non-age-matched groups, which reported a significant effect of BVP on dropout rates. The other similar previous study [17], that did match the ages at group level, also reported more dropout of participants with BVP, though not statistically analyzed. Here, with age- and sex-matching, we did not find an effect of BVP on dropout rate in either the planned or post hoc analyses, suggesting that the previous results were, at least in part, attributable to age, sex, or other differences rather than BVP itself. Therefore, walking capacity, particularly the ability to walk at faster speeds, is not disproportionately impaired in individuals with BVP beyond the effects of normal aging. Although people with BVP are often thought to struggle with speeds above 5 km/h [32], only 6 of 41 participants (eTable S2 in Supplement 1) with BVP dropped out at this speed, nearly matching the dropout rate of the healthy group and this was similar in the no handrail use group (eTable S5 in Supplement 1). Due to the small sample size after excluding those who used the handrail, neither age nor BVP had an effect on the dropout rate (eTable S4 in Supplement 1). As highlighted in McCrum [33], who examined gait variability at a range of walking speeds (0.4–1.6 m/s), most participants who dropped out at increasing speeds did so because they were unable to maintain the required walking speed, rather than due to imbalance. This suggests that physical fitness and walking ability may play a significant role in determining whether BVP patients or healthy adults can complete assessments at higher speeds without use of the handrails.

We hypothesized that participants with BVP would exhibit greater DVAL compared to healthy age–sex-matched participants, and this hypothesis was confirmed by the results. Consistent with previous work [19], DVAL was lower in participants with BVP across all walking speeds (*p* < 0.001). Moreover, in participants with BVP, walking speed was the only factor that significantly influenced DVAL (*p* < 0.001). The analysis without handrail use showed that both group and speed affected DVAL. Unlike dropout rates, which may be influenced by physical capacity, DVAL is a more specific measure of vestibular function. Therefore, it is unsurprising that only participants with BVP experienced a decline in DVA, particularly as head accelerations and displacement increase at faster walking speeds [34] and that greater head movement amplitude and frequency place additional strain on DVA [21, 35].

We found significant differences in DVAL depending on whether participants with BVP held the handrails. At speeds of 4 km/h (*p* = 0.003) and 6 km/h (*p* = 0.017), participants who used the handrails experienced less DVAL compared to those who did not. This indicates that handrail support may provide stabilization to the neck and head, via the arms and shoulders, allowing better DVA at higher speeds. Additionally, it could be that handrail use not only provided direct stabilization but also additional proprioceptive information that benefitted postural control, self-motion perception and head stabilization during walking [36–38]. At 2 km/h, the need for precise head stabilization is less critical; there were no group differences in DVAL. In the post hoc analysis of the BVP group without handrail support, the interaction between group and speed was not significant for DVAL, but both group and speed had a significant effect on DVAL and eFigure S2 in Supplement 1 appears to show an interaction that we were perhaps no longer powered to detect. We conducted additional post hoc analyses of the associations between the DVAL and the three vestibular tests in our participants with BVP but found no evidence for a clear relationship between DVAL and the extent of vestibular loss (eResults in Supplement 1). Overall, the differential response in DVAL between groups highlights the importance of understanding how BVP and walking speed interact and may help inform the tailoring of vestibular rehabilitation programs. Interventions that progressively challenge patients at various walking speeds may help patients develop strategies to cope with dynamic situations.

This study relied on a questionnaire to confirm the absence of vestibular or other disorders in the healthy group. This limitation suggests that group differences on dropout rates and DVA may have been slightly underestimated. Another general limitation of our DVA testing approach is that fixed speeds are used, as opposed to participants’ preferred or maximal walking speeds, which may result in more complete data, though not then standardized. A potential addition in future protocols could be to use a dimensionless walking speed which would account for anatomical differences in leg length or height [39]. However, more research on these additional conditions is needed before clear recommendations for clinical practice can be made, since it is currently unclear how individualized walking speeds will affect the standardization of the DVA outcomes and how these should then be interpreted.

In conclusion, based on our comparison of participants with BVP and healthy age–sex-matched participants, BVP does not appear to restrict the ability to walk at the higher speeds of a DVA assessment and cause an increase in dropout rate, whereas age does. BVP significantly impacts DVA, with increasing DVAL at increasing walking speeds.

## Supplementary Information

Below is the link to the electronic supplementary material.

Supplementary file1 (PDF 338 KB)

## Data Availability

Anonymized DVAL and dropout data and SPSS syntax for the statistical analyses are available on the Open Science Framework at https://osf.io/b6usg/
