# Supplementary material for: Dynamic visual acuity in bilateral vestibulopathy and healthy age–sex-matched participants
Source: J Neurol. 2025 Sep 1;272(9):603. doi: 10.1007/s00415-025-13269-9 (PMC12401756; doi:10.1007/s00415-025-13269-9)
Supplement: Supplementary file 1 — Supplementary file1 (PDF 338 KB) [file 415_2025_13269_MOESM1_ESM.pdf]

Supplemental Online Content for:

Dynamic visual acuity in bilateral vestibulopathy and healthy age-sex-matched participants

Meichan Zhu[1,2,3*], Lisa van Stiphout[1], Benjamin Volpe[1], Miranda Janssen[1,4], Mustafa Karabulut[1], Angélica Pérez Fornos[5], Nils Guinand[5], Kenneth Meijer[2], Raymond van de Berg[1], Christopher McCrum[2*]

[1]Department of Otorhinolaryngology and Head and Neck Surgery, Division of Balance Disorders, Maastricht University Medical Center, School for Mental Health and Neuroscience, Maastricht, The Netherlands
[2]Department of Nutrition and Movement Sciences, NUTRIM Institute of Nutrition and Translational Research in Metabolism, Maastricht University, Maastricht, The Netherlands
[3]Department of Otorhinolaryngology, Guangzhou Twelfth People's Hospital (Guangzhou Otolarynology-Head and Neck Surgery Hospital), No. 1 Tianqiang Road, Tianhe District, Guangzhou, 510620, Guangdong, China.
[4]Department of Methodology and Statistics, Care and Public Health Research Institute (CAPHRI), Maastricht University, Maastricht, The Netherlands
[5]Service of Otorhinolaryngology and Head and Neck Surgery, Department of Clinical Neurosciences, Geneva University Hospitals, Geneva, Switzerland

*Correspondence:
Meichan Zhu: z.meichan@maastrichtuniversity.nl
Christopher McCrum: chris.mccrum@maastrichtuniversity.nl

Content:

eMethods: Assessing vestibular function in participants with BVP
eResults: BVP Etiologies
eTable 1: Descriptives of BVP vHIT, caloric test, torsion swing test
eTable 2: Decade of drop out
eResults: Post hoc analysis ecluding participants using the handrails
eFigure1: DVAL at the three walking speeds in participants with no handrail BVP (green) and healthy age-sex-matched participants (blue)
eTable 3. Participant demographic data
eTable 4. Drop out rate BVP patients without handrail use
eTable 5. Decade of drop out
eResults: Dynamic visual acuity loss (without using the handrail
eTable 6. Main factor results of DVAL model
eTable 7. main factor results of final DVAL model building
eResults: Post hoc analysis of associations between vestibular tests and DVAL
eTable 8. Correlation of vestibular test (vHIT, caloric chair and torsion swing test) and DVAL
eTable 9. Correlation of vestibular test (vHIT, caloric chair and torsion swing test) and DVAL
eReferences

eMethods

Assessing vestibular function in participants with BVP

Once enrolled in the study, the participants' diagnoses of BVP was confirmed on the day of the measurements, since some participants' diagnoses were evaluated some time before the study. This was done using the results from three tests in accordance with the Bárány criteria[1,2] as follows: imbalance and/or oscillopsia during walking or head movements, along with a reduced bithermal caloric response (total bithermal maximal peak slow-phase velocity <6°/s bilaterally at 30 and 44 °C, 300 ml in 30 s) and/or a bilaterally reduced video-head impulse test (vHIT) gain of <0.6, and/or a VOR gain ≤0.1 during the torsion swing test. The horizontal vHIT, along with the vHIT in the Right-Anterior-Left-Posterior (RALP) and Left-Anterior-Right-Posterior (LARP) canal planes, was conducted using the vHIT[3,4] with the vHIT device from Otometrics (Otometrics, Taastrup, Denmark). The testing procedure has been previously described[5,6]. Briefly, the assessor stood behind the seated participant, firmly holding their head without touching the goggles. The participant was instructed to maintain visual fixation on a stationary target positioned 2 meters away. Head impulses consisted of rapid, unpredictable, low-amplitude (±20°) movements in the horizontal plane (peak velocity >150°/s) and in the RALP and LARP planes (peak velocity >100°/s). The Otometrics system calculated the VOR gain as the ratio of the area under the eye velocity curve to the area under the head velocity curve, measured from the onset of the impulse until the head velocity returns to zero[3]. Bithermal caloric testing[7] was conducted on both ears while participants were in a supine position with their heads inclined forward at 30°. Each irrigation lasted 30 seconds, using at least 250 mL of water at temperatures of 30°C (cold) and 44°C (warm), with a 5-minute interval between stimulations (Variotherm Plus device, Lenzkirch, German). Eye movements were recorded via electronystagmography using self-adhesive electrodes (Blue Sensor, Ambu, Denmark). The maximum peak slow-phase eye velocity at the culmination phase (°/s) was measured (KingsLab 1.8.1, Maastricht University, Maastricht, The Netherlands). Finally, the torsion swing test was conducted with participants seated in a servo-controlled rotatory chair in complete darkness with their eyes open (Ekida GmbH, Buggingen, Germany). Sinusoidal rotatory stimulation was applied at a frequency of 0.1 Hz with a peak velocity of 60°/s. Eye movements were recorded using electronystagmography with self-adhesive electrodes (Blue Sensor, Ambu, Denmark). The vestibulo-ocular reflex (VOR) gain was determined as the ratio of peak eye velocity to peak head velocity (KingsLab 1.8.1, Maastricht University, Maastricht, The Netherlands)[8,9].

eResults

BVP Etiologies

For the etiology of 41 cases of BVP, the distribution was as follows: idiopathic (n=18, of which 6 cases associated with migraine); genetic (n=7); ototoxic (n=7); auto-immune (n=6); meningitis and radiotherapy (n=1); other ear pathology (n=3, of which 1 case with Menière's Disease, and 2 cases with bilateral labyrinthitis); neurodegenerative related disease (n=1).

eTable 1. Descriptives of BVP vHIT ,caloric test, torsion swing test (n=41)

	vHIT_ gain_RL	vHIT_ gain_LL	vHIT_ gain_RP	vHIT_ gain_LA	vHIT_ gain_RA	vHIT_ gain_LP	Caloric right (°/s)	Caloric left(°/s)	Torsion swing gain(%)	Torsion swing phase (°)
**Mean**	0.31	0.31	0.21	0.36	0.38	0.30	1.44	1.93	3.76	47.24
**Median**	0.25	0.25	0.17	0.28	0.38	0.25				
**Standard deviation**	0.20	0.25	0.15	0.22	0.20	0.19	2.73	4.15	4.14	152.76
**Minimum**	-0.01	-0.18	0.03	0.09	0.00	0.03	0	0		
**Maximum**	0.89	0.95	0.60	0.91	0.82	0.93	11	20		

vHIT gains: for the leftward and rightward directions in the lateral plane, as well as for the upward and downward directions in right anterior—left posterior and left anterior—right posterior planes.
The caloric test's: outcome: sum of bithermal max peak slow phase caloric induced nystagmus
The torsion swing test was conducted by the velocity-controlled rotatory chair. Outcome parameters are VOR gain.

eTable 2. Decade of drop out (n=41)

			Decade of Age (y)					
			19-29	30-39	40-49	50-59	60-69	70-79
**BVP**	n		1	2	5	15	11	7
	Dropout (n)	4km/h & 6km/h	0	0	0	0	0	1
		6km/h	0	0	0	1	2	3
**Healthy**	n		1	3	4	12	14	7
	Dropout (n)	4km/h & 6km/h	0	0	0	0	0	1
		6km/h	0	0	0	1	3	1

Post hoc analysis excluding participants using the handrails

Due to the significant impact of handrail use on DVAL, we decided to repeat our analyses with only the participants with BVP who did not use the handrails and their corresponding healthy age-sex-matched participants. Briefly, the re-analysis of dropout rate was limited by the fact that only one participant dropped out (a healthy participant at 6km/h), though this confirms our previous result of no effect of BVP on dropout, since no participant with BVP in this analysis dropped out. Contrary to the previous analysis, age was not found to be a significant factor, probably due to the fact that the re-analysis was underpowered, since only one participant dropped out. The re-analysis of DVAL followed the same model building process, as described in the statistical analyses section, and resulted in a marginal model with unstructured covariance matrix of the residuals. No significant Group*Speed interaction effect on DVAL was observed ($F_{2,43.84}$=1.204, p=0.310) and no effect of age on DVAL was found ($F_{1,40.73}$=0.057, p=0.813). Following the top-down procedure, these terms were removed from the model and the final model found significant effects of group ($F_{1,42.85}$=51.35, p<0.001) and speed ($F_{1,41.33}$=5.30, p=0.009) on DVAL (eFigure 1).

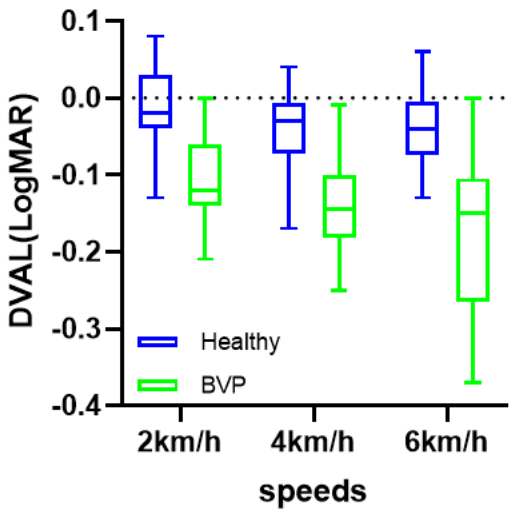

eFigure 1. DVAL at the three walking speeds in participants with no handrail BVP (green) and healthy age-sex-matched participants (blue). Box: lower quartile, median, upper quartile. Whiskers: minimum and maximum values.

eTable 3. Participant demographic data (n=22)

	BVP	Range	N	Healthy Control	N	Range	t(df)	P
**Age, years**	49.9(9.8)	25-62	22	49.4(9.8)	22	25-62	t(42) = 0.18	p=0.855
**Male (%)**	59.09%		13	59.09%	13			
**Height, cm**	174.3(10.0)	152.0-196.0	22	173.7(11.5)	22	158.0-202.5	t(42) = 0.16	P=0.875
**Body weight, kg**	79.7(21.2)	40.1-130.0	22	74.3(11.5)	22	60.0-104.0	t(42) = 1.06	P=0.297
**BMI**	25.9(5.0)	17.4-36.8	22	24.6(2.8)	22	19.3-29.6	t(42) = 1.05	P=0.298

eTable 4. Drop out rate BVP patients without handrail use (n=22)

factor	B	SE	df	P
**age**	0.091	0.159	1	0.565
**group**	18.162	8332.366	1	0.998

eTable 5. Decade of drop out (n=22)

			Decade of Age (y)					
			19-29	30-39	40-49	50-59	60-69	70-79
**BVP**	n		1	2	5	12	2	0
	Dropout (n)	4km/h & 6km/h	0	0	0	0	0	1
		6km/h	0	0	0	0	0	0
**Healthy**	n		1	3	4	10	4	0
	Dropout (n)	4km/h & 6km/h	0	0	0	1	0	0
		6km/h	0	0	0	0	0	0

Dynamic visual acuity loss (without using the handrail ,n=22)

The model building, as described in the statistical analyses section, resulted in a marginal model with unstructured covariance matrix of the residuals. DVA loss was the outcome at any given speed point in both groups. Main effects included group, age, speed and an interaction between group and speed (Group*Speed). This revealed no significant Group*Speed interaction effect ($F_{2,43.84}$=1.204, p=0.310) on DVA loss. Age did not have a significant effect on the DVA loss ($F_{1,40.73}$=0.057, p=0.813). Top-down procedure was performed in the model building. Group ($F_{1,42.85}$=51.35, p<0.001) is significantly different in DVAL, and speed ($F_{1,41.33}$=5.30, p=0.009) as well.

eTable 6. Main factor results of DVAL model (n=22)

factor	Numerator df	Denominator df	F	p
**group**	1	40.855	48.564	.000
**speed**	2	43.214	.206	.815
**age**	1	40.727	.057	.813
**group *speed**	2	43.836	1.204	.310
**speed * age**	2	43.471	.701	.502

eTable 7. main factor results of final DVAL model building (n=22)

factor	Numerator df	Denominator df	F	p
**group**	1	42.847	51.346	.000
**speed**	2	41.330	5.301	.009

Post hoc analysis of associations between vestibular tests and DVAL

Our results indicate different effects of age-related differences and differences related to the presence of BVP. To further explore and better understand our results, we conducted some additional exploratory analyses on the associations between the DVAL in our participants with BVP, and their test results on the vHIT, caloric test, and torsion swing test to explore if there was an association with the severity of vestibular loss. To do this, we performed Pearson correlations between all vestibular test scores and DVAL at all three speeds, for both the complete BVP data set and for the no handrail use data set. Supplement Tables 8 and 9 display the 60 correlations run (30 for all 41 participants and 30 for the 22 participants who did not use the handrails), only 5 were significant (approximately in alignment with the false-positive rate) and only 1 vestibular test outcome was significantly correlated with the DVAL in both samples (torsion swing phase and DVAL at 6km/h). Therefore, we did not observe any evidence for a strong relationship between DVAL and the extent of vestibular loss.

eTable 8. Correlation of vestibular test (vHIT, caloric chair and torsion swing test) and DVAL (n=41)

		2km	4km	6km
**vHIT_gain_LL**	Pearson's r	-0.04	-0.02	0.21
	p-value	0.828	0.912	0.245
**vHIT_gain_RP**	Pearson's r	0.02	-0.09	0.25
	p-value	0.918	0.578	0.165
**vHIT_gain_LA**	Pearson's r	0.12	0.19	0.43
	p-value	0.445	0.252	0.013
**vHIT_gain_RA**	Pearson's r	0.07	-0.01	0.20
	p-value	0.680	0.952	0.253
**vHIT_gain_LP**	Pearson's r	0.05	-0.11	0.18
	p-value	0.758	0.485	0.307
**vHIT_gain_RL**	Pearson's r	0.13	-0.01	0.26
	p-value	0.423	0.971	0.142
**CALORIC_LEFT_SUM**	Pearson's r	0.00	0.08	0.14
	p-value	0.977	0.637	0.435
**CALORIC_RIGHT_SUM**	Pearson's r	0.19	0.16	0.13
	p-value	0.240	0.322	0.476
**TORSION_SWING_PHASE_(°)**	Pearson's r	-0.05	-0.06	0.20
	p-value	0.740	0.703	0.262
**TORSION_SWING_GAIN_(%)**	Pearson's r	0.14	0.26	0.35
	p-value	0.378	0.106	0.046

eTable 9. Correlation of vestibular test (vHIT, caloric chair and torsion swing test) and DVAL (n=22)

		2km	4km	6km
**vHIT_gain_LL**	Pearson's r	0.02	0.05	0.14
	p-value	0.937	0.817	0.546
**vHIT_gain_RP**	Pearson's r	-0.16	-0.10	0.27
	p-value	0.464	0.673	0.228
**vHIT_gain_LA**	Pearson's r	0.16	0.21	0.30
	p-value	0.464	0.348	0.184
**vHIT_gain_RA**	Pearson's r	-0.02	-0.03	0.09
	p-value	0.927	0.901	0.711
**vHIT_gain_LP**	Pearson's r	-0.15	-0.15	0.14
	p-value	0.499	0.503	0.537
**vHIT_gain_RL**	Pearson's r	0.05	0.06	0.20
	p-value	0.814	0.786	0.388
**CALORIC_RIGHT_SUM**	Pearson's r	0.00	0.32	0.07
	p-value	0.992	0.153	0.777
**CALORIC_LEFT_SUM**	Pearson's r	-0.13	0.13	0.05
	p-value	0.578	0.554	0.815
**TORSION_SWING_GAIN_(%)**	Pearson's r	0.15	0.43	0.23
	p-value	0.501	0.044	0.308
**TORSION_SWING_PHASE_(°)**	Pearson's r	0.17	0.43	0.45
	p-value	0.446	0.048	0.042

eReferences

1. Strupp M, Kim JS, Murofushi T, et al. Bilateral vestibulopathy: Diagnostic criteria Consensus document of the Classification Committee of the Barany Society. *Journal of vestibular research : equilibrium & orientation*. 2017;27(4):177-189. doi:10.3233/VES-170619
2. Strupp M, Kim JS, Murofushi T, et al. Erratum to: Bilateral vestibulopathy: Diagnostic criteria Consensus document of the Classification Committee of the Barany Society. *J Vestib Res*. 2023;33(1):87. doi:10.3233/VES-229002
3. Macdougall HG, McGarvie LA, Halmagyi GM, Curthoys IS, Weber KP. The video Head Impulse Test (vHIT) detects vertical semicircular canal dysfunction. *PloS one*. 2013;8(4):e61488. doi:10.1371/journal.pone.0061488
4. MacDougall HG, Weber KP, McGarvie LA, Halmagyi GM, Curthoys IS. The video head impulse test: diagnostic accuracy in peripheral vestibulopathy. *Neurology*. Oct 6 2009;73(14):1134-41. doi:10.1212/WNL.0b013e3181bacf85
5. van Dooren TS, Lucieer FMP, Janssen AML, Kingma H, van de Berg R. The Video Head Impulse Test and the Influence of Daily Use of Spectacles to Correct a Refractive Error. *Frontiers in neurology*. 2018;9:125. doi:10.3389/fneur.2018.00125
6. van Dooren TS, Starkov D, Lucieer FMP, et al. Comparison of three video head impulse test systems for the diagnosis of bilateral vestibulopathy. *Journal of neurology*. Dec 2020;267(Suppl 1):256-264. doi:10.1007/s00415-020-10060-w
7. Shepard NT, Jacobson GP. The caloric irrigation test. *Handb Clin Neurol*. 2016;137:119-31. doi:10.1016/b978-0-444-63437-5.00009-1
8. Lucieer F, Vonk P, Guinand N, Stokroos R, Kingma H, van de Berg R. Bilateral Vestibular Hypofunction: Insights in Etiologies, Clinical Subtypes, and Diagnostics. *Frontiers in neurology*. 2016;7:26. doi:10.3389/fneur.2016.00026
9. van Stiphout L, Lucieer F, Pleshkov M, et al. Bilateral vestibulopathy decreases self-motion perception. *Journal of neurology*. Oct 2022;269(10):5216-5228. doi:10.1007/s00415-021-10695-3
